# Increasing *Fusobacterium* infections with *Fusobacterium varium*, an emerging pathogen

**DOI:** 10.1371/journal.pone.0266610

**Published:** 2022-04-14

**Authors:** Se Ju Lee, Yae Jee Baek, Jin Nam Kim, Ki Hyun Lee, Eun Hwa Lee, Joon Sup Yeom, Jun Yong Choi, Nam Su Ku, Jin Young Ahn, Jung Ho Kim, Su Jin Jeong

**Affiliations:** Division of Infectious Diseases, Department of Internal Medicine and AIDS Research Institute, Seoul, South Korea; University of North Dakota, UNITED STATES

## Abstract

Infections caused by *Fusobacterium* species are rare; however serious infections with complications or mortality may occur occasionally. We conducted a retrospective study to investigate the clinical features of patients with *Fusobacterium* infections and the differences between infections caused by the species *F*. *necrophorum*, *F*. *nucleatum*, and *F*. *varium*. Additionally, we attempted to identify risk factors for *Fusobacterium*-associated mortality. This study included all patients at a large tertiary care teaching hospital in South Korea with *Fusobacterium* infections from January 2006 to April 2021. Demographic, clinical, laboratory, and outcome data were analyzed. Multiple logistic regression analysis was performed to assess the risk factors for in-hospital mortality associated with *F*. *nucleatum* and *F*. *varium* infections. We identified 272 patients with *Fusobacterium* infections during the study period. The number of *Fusobacterium* cases has increased recently, with *F*. *varium* infections markedly increasing since 2016 and causing a significant proportion of infections. Patients with *F*. *varium* infections were older and had a higher proportion of nosocomial infections than the other groups. The *F*. *nucleatum* and *F*. *varium* groups showed higher in-hospital mortality than the *F*. *necrophorum* group. Through logistic regression analysis, APACHE II score and serum albumin level were considered risk factors for in-hospital mortality. APACHE II score was positively correlated with age, red cell distribution width, and serum blood urea nitrogen, and negatively correlated with serum albumin level. Infections caused by *Fusobacterium* species are increasing. *F*. *varium* causes a significant proportion of severe infections.

## Introduction

*Fusobacterium* is gram-negative anaerobic bacteria usually found as normal flora in the oral cavity and gastrointestinal tract. Recently, *Fusobacterium nucleatum* has been discovered in colorectal cancer tissue, and several studies have reported its relationship with colorectal tumorigenesis [1,2]. The pathogenic role of *F*. *nucleatum* in adverse pregnancy outcomes has also been described previously [3]. Moreover, a link between Crohn`s disease, ulcerative colitis, and *Fusobacterium* has been suggested [4,5]. Infections caused by *Fusobacterium* species are rare, but sometimes serious infections with complications or mortality do occur. Lemierre`s syndrome, which is characterized by thrombophlebitis of the internal jugular vein following oropharyngeal infection, is the representative disease of *Fusobacterium* infection. This syndrome is considered to be “forgotten” due to its low incidence in the post-antibiotic era [6]. However, the number of reported cases has been increasing recently without clear reason [7]. Furthermore, several studies have shown evidence of the growing number of cases of anaerobic infection with *Fusobacterium* species [8]. Fusobacterium infection shows various clinical features, ranging from mild cases to invasive and fatal disease [9,10]. In general, *F*. *necrophorum*, which is known to cause Lemierre`s syndrome, and *F*. *nucleatum* are the two most common species reported [11–13]. However, only a few studies have described the overall characteristics of *Fusobacterium* infection and the differences among species. Accordingly, we conducted a retrospective study to investigate the clinical features of patients with *Fusobacterium* infections and the differences between the infection caused by *F*. *necrophorum*, *F*. *nucleatum*, and *F*. *varium*. We also sought to identify the risk factors for *Fusobacterium* infection-related mortality.

## Materials and methods

### Study design and patient population

This retrospective study included all patients with *Fusobacterium* infections admitted to Severance Hospital, a large tertiary care teaching hospital in South Korea, from January 2006 to April 2021. *Fusobacterium* infection was defined as isolation of *Fusobacterium* species from any patient exhibiting signs of infection. All isolates were identified using either conventional methods [14], Vitek Anaerobe and Corynebacterium identification cards (bioMérieux), or a VITEK MS (bioMérieux) matrix-assisted laser desorption ionization-time of flight mass spectrometry (MALDI-TOF MS) system. Infections were divided into three groups: *F*. *nucleatum*, *F*. *necrophorum*, and *F*. *varium*, which were the most common organisms involved. Other *Fusobacterium* species such as *F*. *mortiferum*, *F*. *ulcerans*, *F*. *necrogenes*, and *F*. *naviforme* were also identified, but they were excluded from this study because of the small number of cases involved. All relevant clinical and laboratory data were collected via electronic medical records to investigate the clinical characteristics of infections.

### Variables

The primary outcome examined was in-hospital mortality. Secondary outcomes included intensive care unit (ICU) admission, readmission within 30 days, length of hospital stay, hemodynamic instability, respiratory failure, acute kidney injury (AKI) requiring renal replacement therapy, duration of antibiotics treatment, cases requiring intervention or surgery, and thrombophlebitis.

The Charlson Comorbidity Index was calculated at admission to classify patients according to their overall comorbidity level [15]. The Sequential Organ Failure Assessment (SOFA) and Acute Physiology and Chronic Health Evaluation (APACHE) II scores were used to measure patients’ severity of illness.

### Definitions

Community-acquired infection was defined as when the infection occurred prior to admission or within 48 h of hospitalization. Polymicrobial infection was defined as when one or more additional bacterial species were isolated from the same individual. Hemodynamic instability was defined by either mean arterial pressure <65 mmHg or requiring vasopressors to maintain a mean arterial pressure >65 mmHg. Patients with ventilator care for hypoxemic or hypercapnic respiratory failure were defined as those experiencing respiratory failure. AKI was defined according to Kidney Disease: Improving Global Outcomes criteria [16].

### Statistical analyses

Patient characteristics and outcomes were assessed between the three species using one-way ANOVA or the Kruskal–Wallis test for continuous variables and the chi-squared test for categorical variables. Continuous variables were checked for normality via the Shapiro–Wilk test. The chi-squared test or Fisher’s exact test were used to find differences in categorical variables between those with *Fusobacterium* infections who survived and those who died. Pearson correlation analysis was performed to investigate the relationships between APACHE II score and other variables. Multiple logistic regression analysis with backward stepwise selection was performed to assess the risk factors for in-hospital mortality of *F*. *nucleatum* and *F*. *varium* infections. *F*. *necrophorum* was not included for analyzing the risk factors of in-hospital mortality as infections by *F*. *necrophorum* are not associated with significant mortality [11,12]. A p-value of <0.05 was considered statistically significant. Statistical analyses were performed using R V.4.0.5 (The R Foundation for Statistical Computing, Vienna, Austria).

The institutional review board of the Yonsei University Health System Clinical Trial Center approved this study and the need for informed consent was waived by the institutional review board because of the study design.

## Results

### Patient characteristics

We identified a total of 272 patients with *Fusobacterium* infections during the study period. Among the 272 patients, there were 86, 71, and 56 cases of *F*. *nucleatum*, *F*. *necrophorum*, and *F*. *varium* infections, respectively. Table 1 presents a comparison of patient characteristics of each group. Patient characteristics were also compared between the groups as follows: *F*. *varium* with *F*. *nucleatum* and *F*. *varium* with *F*. *necrophorum*. The differences between the three groups are shown in Table 1. Patients with *F*. *necrophorum* infections were younger than those in other groups and most cases were less severe community-acquired infections with fewer comorbidities. In contrast, patients in the *F*. *varium* group were older and had a higher proportion of nosocomial infections than those in other groups. There were also differences in infection sites. The majority of *F*. *necrophorum* infections were upper respiratory tract infections, whereas most cases of *F*. *varium* infections were intra-abdominal. Unlike the other two species, *F*. *nucleatum* showed similar rates of upper respiratory infection, intra-abdominal infection, and primary bacteremia.

**Table 1:** Comparison of clinical characteristics of each species.

	*Fusobacterium nucleatum* (n = 86)	*Fusobacterium necrophorum* (n = 71)	*Fusobacterium varium* (n = 56)	p-value	p’	p"
Age, years, median (IQR)	55.5 (39.5–67.5)	27 (22.5–39.0)	62.5 (50.75–69.0)	<0.001	0.115*	<0.001*
Male sex, n (%)	48 (55.8%)	51 (71.8%)	44 (78.6%)	0.011	0.009	0.507
Community acquired, n (%)	59 (68.6%)	63 (88.7%)	19 (33.9%)	<0.001	<0.001	<0.001
Comorbidities, n (%)						
•Hypertension	26 (30.2%)	6 (8.5%)	30 (53.6%)	<0.001	0.009	<0.001
•Diabetes mellitus	12 (14.0%)	0	14 (25.0%)	<0.001	0.149	<0.001
•Congestive heart failure	3 (3.5%)	0	0	0.106	0.278^#^	-
•Coronary artery disease	2 (2.3%)	0	8 (14.3%)	0.000	0.014^#^	0.001^#^
•Peripheral arterial occlusive disease	0	0	3 (5.4%)	0.014	0.059^#^	0.083^#^
•Chronic obstruction pulmonary disease	1 (1.2%)	1 (1.4%)	5 (8.9%)	0.022	0.035^#^	0.086^#^
•Chronic kidney disease	2 (2.3%)	0	14 (25.0%)	<0.001	<0.001	<0.001
•Chronic liver disease	9 (10.5%)	3 (4.2%)	5 (8.9%)	0.341	0.99	0.474
•Cerebrovascular accident	3 (3.5%)	1 (1.4%)	2 (3.6%)	0.680	1^#^	0.835^#^
•Connective tissue disease	1 (1.2%)	0	0	0.476	1^#^	-
•Inflammatory bowel disease	0	0	1 (1.8%)	0.278	0.828^#^	0.441^#^
•Solid organ transplantation	2 (2.3%)	0	0	0.225	0.674^#^	-
•Immunosuppressive therapy	3 (3.5%)	0	2 (3.6%)	1	1^#^	0.193^#^
•Solid cancer	24 (27.9%)	8 (11.3%)	28 (50.0%)	<0.001	0.013	<0.001
•Hematologic malignancy	3 (3.5%)	0	3 (5.4%)	0.172	0.68	0.083^#^
Charlson Comorbidity Index, median (IQR)	2.0 (0.0–5.0)	0.0 (0.0–0.0)	4.0 (2.0–6.0)	<0.001	0.034*	<0.001*
Recent surgery history, n (%)	9 (10.5%)	7 (9.9%)	30 (53.6%)	<0.001	<0.001	<0.001
Laboratory data (culture day), median (IQR)						
•White blood cell count, 10^3^/μL	10.8 (6.4–16.1)	15.7 (13.1–18.7)	9.4 (6.5–12.6)	<0.001	0.246	<0.001
•Segmented neutrophil, %	83.0 (71.1–89.2)	82.8 (78.2–85.3)	80.7 (71.4–89.5)	0.583	0.465	0.254
•Lymphocyte count, 10^3^/μL	0.9 (0.6–1.6)	1.6 (1.3–2.0)	0.9 (0.6–1.4)	<0.001	0.851	<0.001
•Red cell distribution width, %	12.9 (12.4–14.9)	12.4 (12.0–13.0)	13.8 (12.8–15.1)	<0.001	0.159	<0.001
•Platelet count, 10^3^/μL	275.5 (179.0–343.0)	285.0 (242.5–324.5)	230.5 (178.5–353.5)	0.229	0.456	0.042
•Blood urea nitrogen mg/dL	13.4 (9.1–23.0)	12.8 (10.1–15.6)	15.6 (11.3–21.4)	0.044	0.335	0.007
•Creatinine, mg/dL	0.7 (0.6–1.0)	0.8 (0.7–0.9)	0.8 (0.6–1.1)	0.733	0.530	0.969
•Albumin, mg/dL	3.5 (2.9–4.0)	4.3 (3.9–4.5)	3.2 (2.8–3.5)	<0.001	0.002	<0.001
•Total bilirubin, mg/dL	0.8 (0.5–1.3)	0.8 (0.6–1.2)	0.7 (0.4–1.2)	0.129	0.060	0.121
•Aspartate aminotransferase, IU/L	28.5 (18.0–45.0)	20.0 (16.0–30.5)	24.5 (17.0–36.5)	0.009	0.259	0.076
•Alanine aminotransferase, IU/L	18.5 (13.0–38.0)	16.0 (11.5–27.0)	21.0 (11.0–40.5)	0.433	0.890	0.369
•International normalized ratio	1.1 (1.0–1.2)	1.1 (1.0–1.2)	1.1 (1.1–1.2)	0.067	0.071	0.021
•C-reactive protein, mg/L	72.7 (31.9–122.8)	72.8 (29.0–137.7)	96.3 (50.8–151.4)	0.547	0.356	0.301
•Erythrocyte sedimentation rate, mm/hr	56.0 (40.0–77.0)	44.0 (32.5–62.0)	56.0 (40.5–72.0)	0.021	0.727	0.014
Severity scale, median (IQR)						
•SOFA	0.0 (0.0–3.0)	0.0 (0.0–0.0)	1.0 (0.0–3.0)	<0.001	0.405*	<0.001*
•APACHE II	7.0 (3.0–11.0)	2.0 (1.0–4.5)	6.5 (4.0–11.0)	<0.001	0.816*	<0.001*
Polymicrobial infection, n (%)	44 (51.2%)	44 (62.0%)	51 (91.1%)	<0.001	<0.001	<0.001
•Common co-pathogens, n (%)						
•α-*streptococcus*	23 (52.3%)	40 (90.9%)	8 (15.7%)			
•*Bacteroides* species	5 (11.4%)	1 (2.3%)	32 (62.7%)			
•*Escherichia coli*	4 (9.1%)	2 (4.5%)	17 (33.3%)			
Infection site, n (%)				<0.001	<0.001	<0.001
•Upper respiratory tract infection	25 (29.1%)	60 (84.5%)	0			
•Intra-abdominal infection	21 (24.4%)	8 (11.3%)	43 (76.8%)			
•Pleuropulmonary infection	4 (4.7%)	0	1 (1.8%)			
•Urinary tract infection	1 (1.2%)	0	0			
•Cardiovascular infection	1 (0.4%)	0	0			
•Central nervous system infection	5 (5.8%)	1 (1.4%)	0			
•Skin and soft tissue infection	7 (8.1%)	0	7 (12.5%)			
•Bond and joint infection	1 (1.2%)	0	1 (1.8%)			
•Reproductive organ infection	0	1 (1.4%)	2 (3.6%)			
•Unspecified bacteremia	21 (24.4%)	1 (1.4%)	2 (3.6%)			

p’, *F*. *varium* and *F*. *nucleatum*; p", *F*. *varium* and *F*. *necrophorum*;

*, Mann Whitney U test;

^#^, Fisher test,

IQR, Interquartile range; SOFA, Sequential Organ Failure Assessment; APACHE, Acute Physiology and Chronic Health Evaluation.

### Year-wise distribution of cases

**Fig 1:**
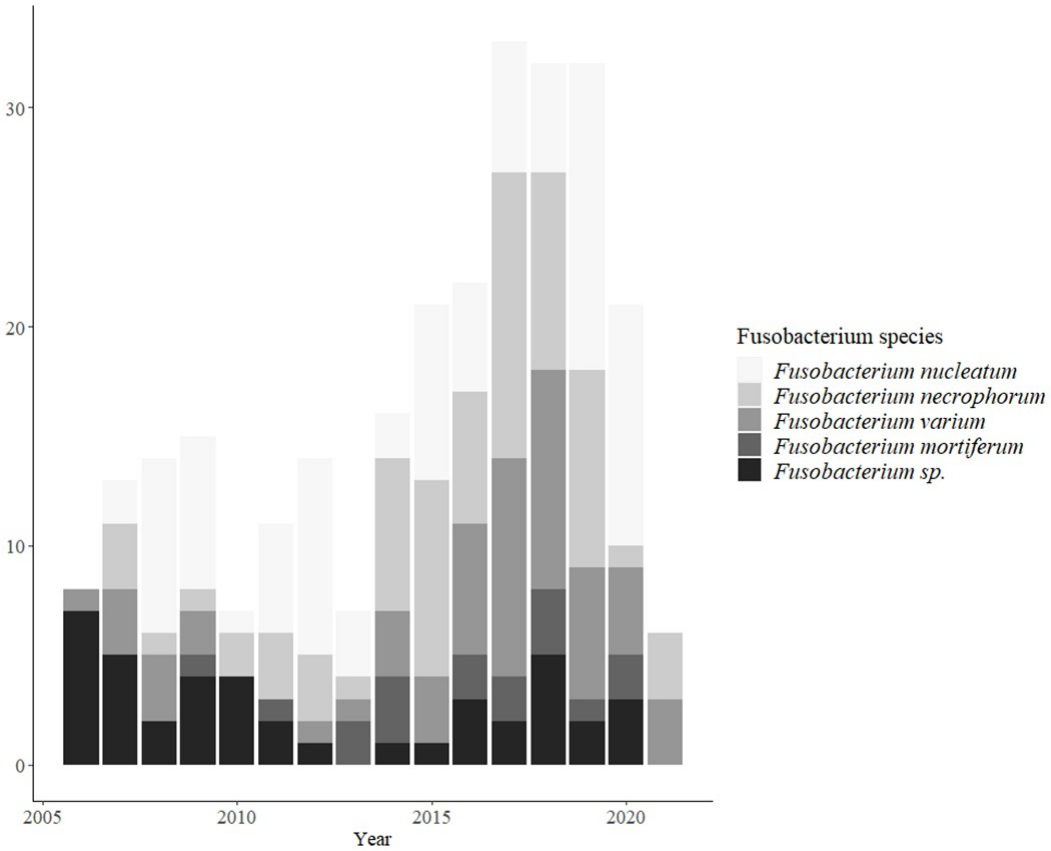
Year-wise distribution of cases and proportion of each species.

The number of *Fusobacterium* infection cases was increased in recent years (Fig 1). *F*. *varium* infections markedly increased and made up a significant proportion of *Fusobacterium* infections from 2016 onwards. Cases of *F*. *nucleatum* and *F*. *necrophorum* infections remained steady throughout the study period.

### Outcomes measured

*The F*. *nucleatum* and *F*. *varium* groups showed markedly higher in-hospital mortality than the *F*. *necrophorum* group (Table 2). Other outcome variables like ICU admission, hemodynamic instability, and respiratory failure also showed similar results. Furthermore, AKI requiring renal replacement therapy occurred more frequently in the *F*. *varium* group. The *F*. *varium* group also had a longer hospital stay and duration of antibiotic therapy than the other groups.

**Table 2:** Outcome analysis of Fusobacterial infections.

	*Fusobacterium nucleatum* (n = 86)	*Fusobacterium necrophorum* (n = 71)	*Fusobacterium varium* (n = 56)	p-value	p’	p"
In-hospital mortality, n (%)	11 (12.8%)	1 (1.4%)	7 (12.5%)	0.025	1	0.021^#^
ICU admission, n (%)	11 (12.8%)	3 (4.2%)	15 (26.8%)	0.001	0.059	0.001
Re-admission in 30days, n (%)	6 (7.0%)	5 (7.0%)	3 (5.4%)	0.913	1^#^	1^#^
Hospital stay, days, median (IQR)	11.0 (2.0–19.0)	4.0 (3.0–6.5)	23.5 (11.0–38.5)	<0.001^+^	<0.001*	<0.001*
Hemodynamic instability, n (%)	18 (20.9%)	3 (4.2%)	12 (21.4%)	0.006	1	0.007
Respiratory failure, n (%)	8 (9.3%)	2 (2.8%)	9 (16.1%)	0.033	0.342	0.011^#^
AKI requiring RRT, n (%)	2 (2.3%)	0	8 (14.3%)	<0.001	0.014^#^	0.001^#^
Duration of antibiotic therapy, days, median (IQR)	14.5 (9.0–23.0)	13.0 (10.0–17.5)	24.0 (14.0–38.5)	0.007^+^	0.001*	<0.001*
Requiring intervention or surgery for treatment, n (%)	54 (62.8%)	65 (91.5%)	49 (87.5%)	<0.001	0.002	0.651
Thrombophlebitis (including Lemierre`s syndrome), n (%)	4 (4.7%)	3 (4.2%)	2 (3.6%)	0.952	1^#^	1^#^

p’, *F*. *varium* and *F*. *nucleatum*; p", *F*. *varium* and *F*. *necrophorum*.

^+^, Kruskal-Wallis test;

*, Mann Whitney U test;

^#^, Fisher test.

IQR, Interquartile range; ICU, Intensive care unit; AKI, Acute kidney injury; RRT, Renal replacement therapy.

### Risk factors for in-hospital mortality in the *F*. *nucleatum* and *F*. *varium* groups

We performed a univariate analysis of the risk factors for in-hospital mortality in the *F*. *nucleatum* and *F*. *varium* groups. As the *F*. *necrophorum* group showed almost no cases of mortality, it was excluded from this analysis (Table 3). Age, Charlson Comorbidity Index, lymphocyte count, platelet count, red cell distribution width (RDW), blood urea nitrogen (BUN), serum albumin, serum total bilirubin, APACHE II score, and SOFA score were significant. When included in logistic regression analysis, Charlson Comorbidity Index and APACHE II scores could be considered as risk factors for in-hospital mortality (Table 4).

**Table 3:** Univariate analysis of risk factors for mortality in the *F*. *nucleatum* and *F*. *varium* groups.

	Survival (n = 124)	Mortality (n = 18)	p-value
Age, years, median (IQR)	55.5 (40.5–67.0)	69.5 (62.0–80.0)	<0.001
Male sex, n (%)	82 (66.1%)	10 (55.6%)	0.539
Community acquired, n (%)	72 (58.1%)	6 (33.3%)	0.086
Charlson Comorbidity Index, median (IQR)	3.0 (0.0–5.0)	7.0 (6.0–8.0)	<0.001
Recent surgery history, n (%)	34 (27.4%)	5 (27.8%)	1
Laboratory data (culture day), median (IQR)			
•Lymphocyte count, 10^3^/μL	1.0 (0.6–1.6)	0.6 (0.4–0.8)	0.002
•Platelet count, 10^3^/μL	269.5 (190.5–348.5)	138.5 (69.0–301.0)	0.002
•Red cell distribution width, %	13.1 (12.4–14.2)	15.8 (14.8–17.6)	<0.001
•Blood urea nitrogen mg/dL	13.5 (9.3–19.9)	32.2 (17.0–47.3)	<0.001
•Albumin, mg/dL	3.5 (3.0–3.9)	2.6 (2.4–2.9)	0.000
•Total bilirubin, mg/dL	0.7 (0.5–1.2)	1.1 (0.7–1.4)	0.017
Severity scale, median (IQR)			
•SOFA	0.0 (0.0–2.0)	5.5 (3.0–9.0)	<0.001
•APACHE II	6.0 (3.0–10.0)	18.0 (11.0–23.0)	<0.001
Infection site, n (%)			0.095
•Upper respiratory tract infection	25 (20.2%)	0	
•Intra-abdominal infection	54 (43.5%)	10 (55.6%)	
•Skin and soft tissue infection	14 (11.3%)	0	
•Pleuropulmonary infection	3 (2.4%)	2 (11.1%)	
•Central nervous system infection	5 (4.0%)	0	
•Reproductive organ infection	2 (1.6%)	0	
•Urinary tract infection	1 (0.8%)	0	
•Cardiovascular infection	1 (0.8%)	0	
•Bone and joint infection	2 (1.6%)	0	
•Unspecified bacteremia	17 (13.7%)	6 (33.3%)	
*Fusobacterium* species, n (%)			1
•*Fusobacterium nucleatum*	75 (60.5%)	11 (61.1%)	
•*Fusobacterium varium*	49 (39.5%)	7 (38.9%)	

IQR, Interquartile range; SOFA, Sequential Organ Failure Assessment; APACHE, Acute Physiology and Chronic Health Evaluation.

**Table 4:** Multivariate analysis of risk factors for mortality, *F*. *nucleatum* and *F*. *varium* groups.

Covariate	OR (95% CI)	p-value
APACHE II	1.12 (1.02–1.25)	0.021
Serum albumin	0.30 (0.09–0.92)	0.045
Blood urea nitrogen	1.01 (0.99–1.05)	0.358
Lymphocyte count	0.66 (0.20–1.32)	0.367
Platelet count	1.00 (0.99–1.00)	0.401
Serum Total bilirubin	0.99 (0.83–1.16)	0.866

OR, Odds ratio; CI, Confidence interval, APACHE, Acute Physiology and Chronic Health Evaluation.

### Correlation between APACHE II score and other variables

**Fig 2:**
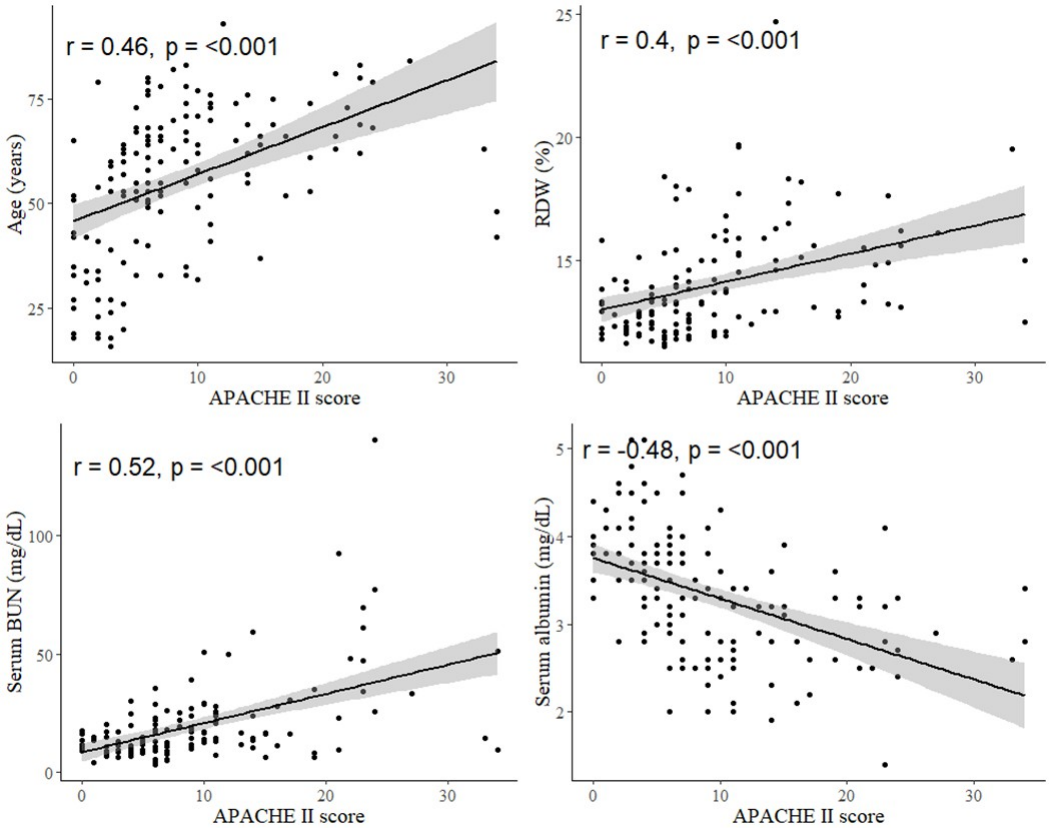
Correlations between APACHE II score and Age, RDW, BUN, and albumin.

The correlation coefficient analysis revealed that age, RDW, and serum BUN were positively associated with the APACHE II score (r = 0.46, 0.4, 0.52, respectively; all p < 0.001). Serum albumin level was negatively correlated with the APACHE II score (r = -0.48, p < 0.001). More details are shown in Fig 2. Multivariate linear regression analysis also identified significant correlations of age, RDW, serum BUN, and serum albumin with APACHE II score.

## Discussion

In this study, we presented the clinical characteristics and outcomes of the three most common species of *Fusobacterium* infection. One of the important findings of our study is that *F*. *varium* infection is serious and as common as infections by the two other known species. *F*. *varium* has gained attention for its potential association with ulcerative colitis; however, infection caused by this species has not been thoroughly investigated and has only been reported in case reports and small series [17–20]. To our knowledge, there has been no study describing the varied clinical characteristics of *F*. *varium* infection. Despite the lack of awareness about this species, surprisingly, *F*. *varium* was found to cause more severe infections than *F*. *nucleatum* and *F*. *necrophorum*. The group of patients with *F*. *varium* showed a high rate of in-hospital mortality (12.5%) as well as a high rate of ICU admission (26.8%) and AKI (14.3%). This is probably because a large proportion of these cases were postoperative nosocomial infections, and these patients had more comorbidities. In addition, the complicated nature of *F*. *varium* infection may be due to the longer resultant hospitalization period and antibiotic treatment when compared with infections caused by the other two species.

Another characteristic of *F*. *varium* infection is a high rate of polymicrobial infections. Consistent with the characteristics of anaerobic infections, polymicrobial infection rates were also high in other groups [21]. Still, the rate of polymicrobial infection was significantly higher in the *F*. *varium* group, and the majority of co-infected pathogens were *Bacteroides* species and *Escherichia coli*, different from the other groups. This might be related to the high rate of intra-abdominal infection in the *F*. *varium* group. Nevertheless, whether this is a unique characteristic of this emerging pathogen requires further research.

As shown in Fig 1, cases with *Fusobacterium* infection have been increasing annually. Among them, the number of *F*. *varium* infections has increased significantly since 2016; however, the reason for this finding is unclear. Most infection sites in the *F*. *varium* group were intra-abdominal, and half of the group had received surgery due to malignant neoplasms like gastric cancer and colorectal cancer. Nevertheless, it is not clear whether the increase of infection by *F*. *varium* is associated with an increased incidence of gastric and colorectal cancer or an increased complication rate after abdominal surgery. Improved diagnostic capability and awareness of this microorganism also can be a reason.

Risk factors for mortality due to *Fusobacterium* infections have not yet been clearly identified. In one study by Su et al., shock, lack of fever at presentation, and underlying diseases (heart failure, renal insufficiency, or malignancy) were presented as independent risk factors for the mortality due to *Fusobacterium* bacteremia [22]. In another study, Yang et al. reported Pitt bacteremia score, nosocomial infection, anemia, ICU stay, renal insufficiency, and hypothermia as risk factors for 30-day mortality in *F*. *nucleatum* bacteremia [23]. Even though the patient population in this study was not equal to that of the previous studies, different results were derived in our study. APACHE II score and serum albumin were identified as risk factors for in-hospital mortality of *F*. *nucleatum* and *F*. *varium* infection. Our results are consistent with previous knowledge considering that APACHE II score is a general marker of critical illness and hypoalbuminemia has a highly significant correlation with mortality [24]. APACHE II score is certainly a useful tool for assessing the severity and prognosis of patients, but it might be cumbersome for routine clinical use. In our study, RDW, serum BUN, and serum albumin level were significantly correlated with APACHE II score. Although these associations with in-hospital mortality were not significant in logistic regression analysis, patients with high RDW or high serum BUN could be considered as severely ill in *F*. *nucleatum* and *F*. *varium* infection.

This study has some limitations. First, the majority of cases in this study were polymicrobial infections, thus the results might not be solely caused by *Fusobacterium* species, but by the accompanying bacteria. However, considering *F*. *nucleatum* serves a structurally supportive role as a bridge organism in dental plaque biofilm, connecting primary colonizers in the oral cavity to anaerobic colonizers, polymicrobial infection might be fundamental to the nature of *Fusobacterium* species [25]. Furthermore, interactions with other microorganisms via adhesion like Aid1, CmpA, RadD, Fap2, and FomA support this assumption. Additionally, we could not collect antimicrobial susceptibility data about isolated *Fusobacterium* species as our institute does not routinely perform antimicrobial susceptibility tests for anaerobes. A more detailed conclusion could be drawn if the results of antimicrobial susceptibility tests and the administered antibiotics were analyzed. Lastly, we cannot exclude that our study was influenced by unknown confounding variables because of the retrospective nature of the study design.

## Conclusion

*Fusobacterium* infections are increasing. A significant proportion of infections is caused by *F*. *varium*, which has not received much attention to date. We found that *F*. *varium* infection is as common as infection by the two other known species, and it is serious. Therefore, further study about *F*. *varium* infection is warranted.

## Supporting information

S1 File Anonymized data set. (XLSX)
