# Correction: Increasing *Fusobacterium* infections with *Fusobacterium varium*, an emerging pathogen

**DOI:** 10.1371/journal.pone.0303711

**Published:** 2024-05-09

**Authors:** Se Ju Lee, Yae Jee Baek, Jin Nam Kim, Ki Hyun Lee, Eun Hwa Lee, Joon Sup Yeom, Jun Yong Choi, Nam Su Ku, Jin Young Ahn, Jung Ho Kim, Su Jin Jeong

There is an error in affiliation of authors. The correct affiliation is: Division of Infectious Diseases, Department of Internal Medicine and AIDS Research Institute, Yonsei University College of Medicine, Seoul, South Korea.
